# An Intraoperative Model for Predicting Survival and Deciding Therapeutic Schedules: A Comprehensive Analysis of Peritoneal Metastasis in Patients With Advanced Gastric Cancer

**DOI:** 10.3389/fonc.2020.550526

**Published:** 2020-09-25

**Authors:** Qi-Yue Chen, Zhi-Yu Liu, Qing Zhong, Wen Jiang, Ya-Jun Zhao, Ping Li, Jia-Bin Wang, Jian-Xian Lin, Jun Lu, Long-Long Cao, Mi Lin, Ru-Hong Tu, Ze-Ning Huang, Ju-Li Lin, Hua-Long Zheng, Si-Jin Que, Chao-Hui Zheng, Chang-Ming Huang, Jian-Wei Xie

**Affiliations:** ^1^Department of Gastric Surgery, Fujian Medical University Union Hospital, Fuzhou, China; ^2^Department of General Surgery, Fujian Medical University Union Hospital, Fuzhou, China; ^3^Division of Life Sciences and Medicine, Department of Gastrointestinal Surgery, The First Affiliated Hospital of University of Science and Technology of China, University of Science and Technology of China, Hefei, China; ^4^Anhui Provincial Hospital Affiliated With Anhui Medical University, Hefei, China; ^5^Key Laboratory of Ministry of Education of Gastrointestinal Cancer, Fujian Medical University, Fuzhou, China

**Keywords:** gastric cancer, peritoneal metastasis, preoperative blood index, prognosis model, therapy

## Abstract

**Background and Objective:** No specialized prognostic model for patients with gastric cancer with peritoneal metastasis (GCPM) exists for intraoperative clinical decision making. This study aims to establish a new prognostic model to provide individual treatment decisions for patients with GCPM.

**Method:** This retrospective analysis included 324 patients with GCPM diagnosed pathologically by laparoscopy from January 2007 to January 2018 who were randomly assigned to different sets (227 in the training set and 97 in the internal validation set). A nomogram was established from preoperative and intraoperative variables determined by a Cox model. The predictive ability and clinical applicability of the PM nomogram (PMN) were compared with the 15th Japanese Classification of Gastric Carcinoma (JCGC) Staging Guidelines for PM (P1abc). Additional external validation was performed using a dataset (*n* = 39) from the First Affiliated Hospital of University of Science and Technology of China.

**Results:** The median survival time was 8 (range, 1–90) months. In the training set, each PMN substage had significantly different survival curves (*P* < 0.001), and the PMN was superior to the P1abc based on the results of time-dependent receiver operating characteristic curve, C-index, Akaike information criterion and likelihood ratio chi-square analyses. In the internal and external validation sets, the PMN was also better than the P1abc in terms of its predictive ability. Of the PMN1 patients, those undergoing palliative resection had better overall survival (OS) than those undergoing exploratory surgery (*P* < 0.05). Among the patients undergoing exploratory surgery, those who received chemotherapy exhibited better OS than those who did not (*P* < 0.05). Among the patients who received palliative resection, only PMN1 patients exhibited better OS following chemotherapy (*P* < 0.05).

**Conclusion:** We developed and validated a simple, specific PM model for patients with GCPM that can predict prognosis well and guide treatment decisions.

## Introduction

Gastric cancer (GC) is the fifth most common malignancy worldwide and ranks third in cancer-related mortality (1). Although the popularity of early screening has led to gradual reduction in the incidence and mortality rates of GC over the past few years, the 5-year survival of advanced GC (AGC) patients is only 10–20% (2, 3). That is mainly because of the Peritoneal metastasis (PM). PM is generally regarded as an incurable systemic disease with a poor prognosis. Its presence in patients with AGC accounts for ~53–66% of GC patients with distant metastasis (4, 5). Scholars from different countries have committed themselves to improving the diagnosis and treatment of such patients and are actively exploring multimodal approaches, including surgery reserved for palliative or cytoreductive purposes and systemic chemotherapy (6–8). A variety of models for the prognosis of GC patients (9, 10) have been established in recent years. However, most of these studies were aimed at early GC or AGC patients without distant metastases. Scholars worldwide have also proposed and developed many staging systems for assessing the prognosis of patients with GC with PM (GCPM) in line with the constantly revised and updated PM grade proposed by the Japanese Classification of Gastric Carcinoma (JCGC) (11); among them the Gilly Stage, the PCI and the P1abc (12–14). Gilly stage combines the maximum nodule diameter, nodule position and morphology. But it indicates not only GC but also other digestive tract cancer. And its definitions for the position of nodule were full of subjectivity. The internationally acknowledged PCI takes into consideration the distribution range and size of peritoneal nodules. It was correlated with the prognosis of GCPM patients to some extent. But it only considers the factors of peritoneal nodule and is not able to complete the comprehensive evaluation. As a simple and special staging, P1abc shows an impressing discriminatory ability but it fails to consider the size of the transferred nodules, which was an important factor for peritoneal nodules. As we can find, a model that can effectively predict the prognosis of patients with GCPM is still lacking.

Despite a few advancements in the preoperative diagnosis and early confirmation of GCPM (15–17), effective prediction has not yet been achieved. Currently, staging laparoscopy and pathological biopsy are still the gold standard for diagnosing peritoneal seeding (18, 19). However, due to individual differences in the degree of transfer, surgeons are still looking for possible methods of choosing and planning appropriate treatment according to various observations of the abdomen during surgery. Since Virchow (20) first discovered the relationship between inflammation and carcinoma, an increasing number of studies have indicated that preoperative blood inflammatory markers are closely related to the survival of cancer patients, and preoperative hematological indexes are also widely used to predict GC patient outcomes (21–25). Because the features of peritoneal lesions alone show poor predictive performance, it is hypothesized that preoperative hematologic indexes can supplement evidence-based medicine to predict the prognosis of GCPM. Therefore, our study combined preoperative imaging, hematological indexes and intraoperative peritoneal nodule characteristics to build an intraoperative model for GCPM that can also suggest appropriate treatment options.

## Materials and Methods

### Study Design and Patients

This retrospective study from the Fujian Medical University Union Hospital (FMUUH) Department of Gastric Surgery included 381 consecutive patients with GCPM confirmed through biopsy between January 2007 and January 2018. The inclusion criteria were as follows: (1) histopathological confirmation of GCPM and (2) diagnostic laparoscopy at our department. The exclusion criteria were as follows: (1) PM accompanied simultaneously by other distant metastases, such as liver, bone or brain metastasis; (2) death caused by severe postoperative complications 30 days after surgery; (3) remnant GC; (4) imaging evidence confirming the existence of PM but no peritoneal nodules identified during surgery (P1x); (5) preoperative chemotherapy; and (6) missing survival data. Finally, 324 cases were analyzed (Supplementary Figure 1). The studies involving human participants were reviewed and approved by the Ethics Committee of FMUUH. Written informed consent from the patients was not required for participation in this study in accordance with the national legislation and the institutional requirements.

### Data Collection

The clinical and pathological data of the patients were obtained from a large-scale prospective database. Routine blood tests were performed within 1 week before surgery. Preoperative imaging studies with contrast were routinely performed following endoscopic and upper gastrointestinal examinations to confirm the tumor location and included chest radiography, computed tomography (CT), ultrasonography of the abdomen, bone scans and positron emission tomography-computed tomography (PET-CT), as required to evaluate the clinical stage. We used the 7th edition of the Union for International Cancer Control classification system to assess the clinical and pathological stage. We evaluated whether patients had occult PM through the preoperative CT images. The definition of occult PM was as follows: nodule lesion that was first diagnosed as negative by CT but later confirmed by laparoscopy (26). The amount of ascites was evaluated by preoperative CT as small and within the pelvic cavity or moderate and beyond the pelvic cavity (27). The distribution of PM nodules was classified according to the video database and electronic surgical records. Referring to the literature (12), 5 and 20 mm were selected as the cutoff values for the maximum nodule diameter. Non-wall nodules were defined as peritoneal nodules confined only to non-abdominal wall locations, such as the stomach, greater omentum, and lesser omentum. A wall nodule was defined as a nodule distributed in the abdominal wall. Nodule morphology included local and diffuse types. The former was defined as each peritoneal nodule isolated from each other and was not connected together, while the latter was defined as densely distributed nodules that were connected into a mass or covered the surface of the abdominal wall (Supplementary Figure 2). Nodule distribution sites consisted of the visceral peritoneum (stomach, greater omentum, lesser omentum, anterior lobe of the transverse colonic membrane, or membrane of the pancreatic surface or spleen, etc.), upper abdominal peritoneum and middle and lower abdominal peritoneum. Based on the number of involved areas, the number of nodule distribution sites ranged from 1 to 3. Overall survival (OS) was defined as the time from surgery until the last follow-up or death. The following treatments were included in this study: (1) exploratory surgery (ES); (2) palliative resection (PR); (3) chemotherapy including systemic chemotherapy or systemic chemotherapy combined with intraperitoneal chemoperfusion; and (4) palliative resection and chemotherapy (PRC). The follow-up deadline was April 2019.

The present study selected the neutrophil-to-lymphocyte ratio (NLR), the platelet-to-lymphocyte ratio (PLR), the prognostic nutritional index (PNI), and carbohydrate antigen 19-9 (CA19-9) for the analysis. The NLR was defined as the absolute neutrophil count divided by the absolute lymphocyte count. The PLR was defined as the absolute platelet count divided by the absolute lymphocyte count. The PNI was calculated as 10 ^*^serum albumin (g/dL) + 0.005 ^*^ total lymphocyte count (per mm^3^).

### Peritoneal Metastasis Staging

The 15th JCGC Staging Guidelines for PM (P1abc) are as follows (14, 28): P1a refers to confirmed tumors confined to the stomach, greater omentum, lesser omentum, anterior lobe of the transverse colonic membrane, or membrane of the pancreatic surface or spleen. P1b refers to confirmed tumors that have spread to the peritoneum of the upper abdomen, namely, the parietal peritoneum close to the umbilical side and the visceral peritoneum close to the cranial transverse colon. P1c refers to a diagnosis with tumor migration to the middle and lower abdominal peritoneum. P1x refers to confirmed PM with an unclear distribution.

### Statistical Analysis

All patients were randomly assigned to the training set (70%) or the internal validation set (30%). Using X-tile software, the optimal cutoff values of the NLR, PLR and PNI were 2, 119, and 40, respectively. Continuous variables were analyzed using Student's *t*-tests or Mann-Whitney U tests, and categorical variables were analyzed using the χ^2^ test or Fisher's exact test. The survival rate was calculated using the Kaplan-Meier method, and the survival rates were compared using the log-rank test. The preoperative and intraoperative variables in the PM nomogram (PMN) were selected from the Cox regression models. Each variable in the nomogram has a corresponding weighted score, and the sum of the weighted scores was associated with OS. Based on the sum of the weighted scores, the patients were grouped into three categories: PMN1: PMN score ≤ 21; PMN2: 21 < PMN score ≤ 30.25; and PMN3: PMN score > 30.25. Decision curves were applied to assess the clinical suitability of the nomogram. Harrell's C-index was used to measure the discriminatory ability of different prognostic systems (29, 30). The likelihood ratio chi-square was calculated with Cox regression to measure homogeneity; a higher likelihood ratio chi-square score indicates better homogeneity (31). We used the Akaike information criterion (AIC) within the Cox regression model to compare the performance of the 2 prognostic systems; smaller AIC values represent better optimistic prognostic stratification (32). We calculated the relative likelihood of two models using the following formula: exp {[AIC (model A) -AIC (model B)]/2}. The relative likelihood represents the probability that model A minimizes information as effectively as model B and could thus be interpreted as a *P*-value for the comparison of both AIC values (33). The Bayesian Information Criterion (BIC) was used to assess the overall prognostic performance of different prognostic systems via bootstrap-resampling analysis (34). Stepwise backward variable removal was applied to the multivariate model to identify the most accurate and parsimonious set of predictors (35). A *P* < 0.05 was statistically significant. External validation was performed using a validation cohort from the First Affiliated Hospital of University of Science and Technology of China (external validation set; *n* = 39; 2014–2018), which satisfied the aforementioned inclusion criteria.

All data were processed using SPSS 20.0 (SPSS Inc. Chicago, IL) and R software (version 3.5.0).

## Results

### Demographic and Clinicopathological Data

Table 1 shows the clinical and pathological data of the 227 patients in the training set and the 97 patients in the internal validation set. Age, sex, occult PM, cT stage, cN stage, tumor location, type of surgery, amount of ascites, nodule maximum diameter, nodule morphology, nodule position, number of nodule distribution sites, chemotherapy, P1abc, NLR, PLR, PNI, and CA19-9 were not significantly different between the training set and internal validation set. The median survival time was 8 months (range, 1–90 months). Supplementary Table 1 shows the clinical and pathological data of 39 patients in the external validation set.

**Table 1:** Clinicopathologic characteristics of the patients.

**Category**	**All patients**	**%**	**Training set**	**%**	**Internal validation set**	**%**	***P*-value**
	**(*n =* 324)**		**(*n =* 227)**		**(*n =* 97)**		
**Age (years)**							0.654
≤ 65	218	67.3	151	66.5	67	69.1	
>65	106	32.7	76	33.5	30	30.9	
**Sex**							0.254
Female	212	65.4	153	67.4	59	60.8	
Male	112	34.6	74	32.6	38	39.2	
**Occult peritoneal metastasis**							0.308
No	163	50.3	110	48.5	53	54.6	
Yes	161	49.7	117	51.5	44	45.4	
**cT**							0.123
cT2-3	43	13.3	30	13.2	13	13.4	
cT4	256	79.0	175	77.1	81	83.5	
cTx	25	7.7	22	9.7	3	3.1	
**cN**							0.292
cNx	41	12.7	32	14.1	9	9.3	
cN0	55	17.0	41	18.1	14	14.4	
cN+	228	70.4	154	67.8	74	76.3	
**Tumor location**							0.936
Upper	36	11.1	24.0	10.6	12.0	12.4	
Middle	97	29.9	70.0	30.8	27.0	27.8	
Lower	142	43.8	99.0	43.6	43.0	44.3	
Overlap	49	15.1	34.0	15.0	15.0	15.5	
**Type of surgery**							0.167
Exploratory surgery	237	73.1	161	70.9	76	78.4	
Palliative Resection	87	26.9	66	29.1	21	21.6	
**Amount of ascites**							0.943
None	125	38.6	88.0	38.8	37.0	38.1	
Small	73	22.5	52.0	22.9	21.0	21.6	
Moderate	126	38.9	87.0	38.3	39.0	40.2	
**Nodule maximum diameter**							0.635
<5 mm	96	29.6	68.0	30.0	28.0	28.9	
5–20 mm	168	51.9	120.0	52.9	48.0	49.5	
>20 mm	60	18.5	39.0	17.2	21.0	21.6	
**Nodule morphology**							0.512
Local	176	54.3	126.0	55.5	50.0	51.5	
Diffuse	148	45.7	101.0	44.5	47.0	48.5	
**Nodule position**							0.322
Non-wall	82	25.3	61.0	26.9	21.0	21.6	
Wall	242	74.7	166.0	73.1	76.0	78.4	
**Number of nodule distribution site**							0.180
1	200	61.7	140.0	61.7	60.0	61.9	
2	82	25.3	62.0	27.3	20.0	20.6	
3	42	13.0	25.0	11.0	17.0	17.5	
**Chemotherapy**							0.173
No	145	44.8	96	42.3	49	50.5	
Yes	179	55.2	131	57.7	48	49.5	
**P1abc**							0.446
P1a	82	25.3	61	26.9	21	21.6	
P1b	112	34.6	74	32.6	38	39.2	
P1c	130	40.1	92	40.5	38	39.2	
**SII**							0.425
≤ 352	33	10.2	21	9.3	12	12.4	
>352	291	89.8	206	90.7	85	87.6	
**NLR**							0.390
≤ 2	132	40.7	89	39.2	43	44.3	
>2	192	59.3	138	60.8	54	55.7	
**PLR**							0.911
≤ 119	49	15.1	34	15.0	15	15.5	
>119	275	84.9	193	85.0	82	84.5	
**PNI**							0.084
≤ 40	88	27.2	68	30.0	20	20.6	
>40	236	72.8	159	70.0	77	79.4	
**AGR**							0.316
≤ 7	207	63.9	149	65.6	58	59.8	
>7	117	36.1	78	34.4	39	40.2	
**CA19-9, U/ml**							0.285
≤ 37	227	70.1	155	68.3	72	74.2	
>37	97	29.9	72	31.7	25	25.8	
**Median survival, months**		8(1-90)					

*SD, standard deviation; ASA, American Society of Anesthesiologists; LN, Lymph node; NLR, Neutrophil-to-lymphocyte ratio; PLR, Platelet-to-lymphocyte ratio; PNI, Prognostic nutritional index; Evaluated by computed tomography: small, within the pelvic cavity; moderate, beyond the pelvic cavity*.

### Prognostic Analysis

Table 2 shows the results of the Cox analysis of the training set. Univariate analysis showed that occult PM, cT stage, ascites, nodule maximum diameter, nodule morphology, nodule position, number of nodule distribution sites, P1abc, NLR, PLR, PNI, cancer antigen 125 (CA-125) and CA19-9 were significant factors for OS (all *P* < 0.05). Further multivariate analysis showed that no occult PM, nodule maximum diameter ≥5 mm, diffuse type, wall nodules, number of nodule distribution sites > 2, high PNI and high CA19-9 levels were independent risk factors for GCPM (*P* < 0.05). Supplementary Table 2 shows the Cox analysis of P1abc for the training set and confirms that the P1abc was an independent risk factor for GCPM (*P* < 0.001).

**Table 2:** Cox regression analysis of the training set of patients with GCPM by preoperative and intraoperative variables.

**Variable**	**Training set (*****n=*** **227)**			
	**Univariate Model**		**Reduced Multivariate Model**	
	**Hazard ratio**	***P***	**Hazard ratio**	***P***
	**(95%CI)**		**(95%CI)**	
**Age (years)**				
≤ 65	Ref			
>65	1.219 (0.915–1.625)	0.175		
**Sex**				
Female	Ref			
Male	1.030 (0.770–1.370)	0.862		
**Occult peritoneal metastasis**				
No	Ref		Ref	
Yes	0.708 (0.540–0.927)	**0.012**	0.673 (0.503–0.903)	**0.008**
**cT**		**0.023**		
cT2-3	Ref			
cT4a	0.728 (0.481–1.101)	0.132		
cT4b	1.018 (0.660–1.568)	0.937		
cTx	1.398 (0.806–2.426)	0.234		
**cN**		0.223		
cN0	Ref			
cN+	1.298 (0.911–1.850)	0.149		
cNx	1.498 (0.918–2.444)	0.106		
**Tumor location**		0.226		
Upper	Ref			
Middle	1.340 (0.831–2.159)	0.230		
Lower	1.524 (0.962–2.412)	0.072		
Overlap	1.127 (0.649–1.956)	0.671		
**Amount of ascites**		**0.004**		
None	Ref			
Small	1.091 (0.760–1.565)	0.637		
Moderate	1.649 (1.213–2.240)	**0.001**		
**Nodule maximum diameter**		** <0.001**		**0.013**
<5 mm	Ref		Ref	
5–20 mm	1.930 (1.409–2.643)	** <0.001**	1.663 (1.172–2.359)	**0.004**
>20 mm	2.615 (1.738–3.934)	** <0.001**	1.633 (1.039–2.566)	**0.034**
**Nodule morphology**				
Local	Ref		Ref	
Diffuse	1.820 (1.380–2.390)	** <0.001**	1.369 (1.005–1.865)	**0.046**
**Nodule position**				
Non-wall	Ref		Ref	
Wall	2.566 (1.865–3.53)	** <0.001**	2.173 (1.509–3.131)	** <0.001**
**Number of nodule distribution site**		**0.030**		**0.007**
1	Ref		Ref	
2	1.388 (1.02–1.889)	**0.037**	1.557 (1.122–2.161)	**0.008**
3	1.403 (0.906–2.174)	0.129	1.706 (1.087–2.679)	**0.020**
**SII**				
≤ 352	Ref			
>352	1.564 (0.982–2.492)	0.059		
**NLR**				
≤ 2	Ref			
>2	1.492 (1.129–1.972)	**0.005**		
**PLR**				
≤ 119	Ref			
>119	1.603 (1.093–2.352)	**0.016**		
**PNI**				
≤ 40	Ref		Ref	
>40	0.665 (0.494–0.895)	**0.007**	0.596 (0.437–0.813)	**0.001**
**AGR**				
≤ 7	Ref			
>7	0.920 (0.691–1.225)	0.568		
**CEA, ng/ml**				
<5	Ref			
≥5	1.317 (0.977–1.775)	0.071		
**CA-125, U/ml**				
<35	Ref			
≥35	1.445 (1.091–1.914)	**0.010**		
**CA19-9, U/ml**				
≤ 37	Ref		Ref	
>37	1.341 (1.004–1.791)	**0.047**	1.626(1.205-2.195)	**0.001**

*A significant collinear relationship between the P1abc and nodule position.

*NLR, Neutrophil-to-lymphocyte ratio; PLR, Platelet-to-lymphocyte ratio; PNI, Prognostic nutritional index; Evaluated by computed tomography: small, within the pelvic cavity; moderate, beyond the pelvic cavity. The bold values are p < 0.05 statistically significant*.

### Establishing the PMN

**Figure 1:**
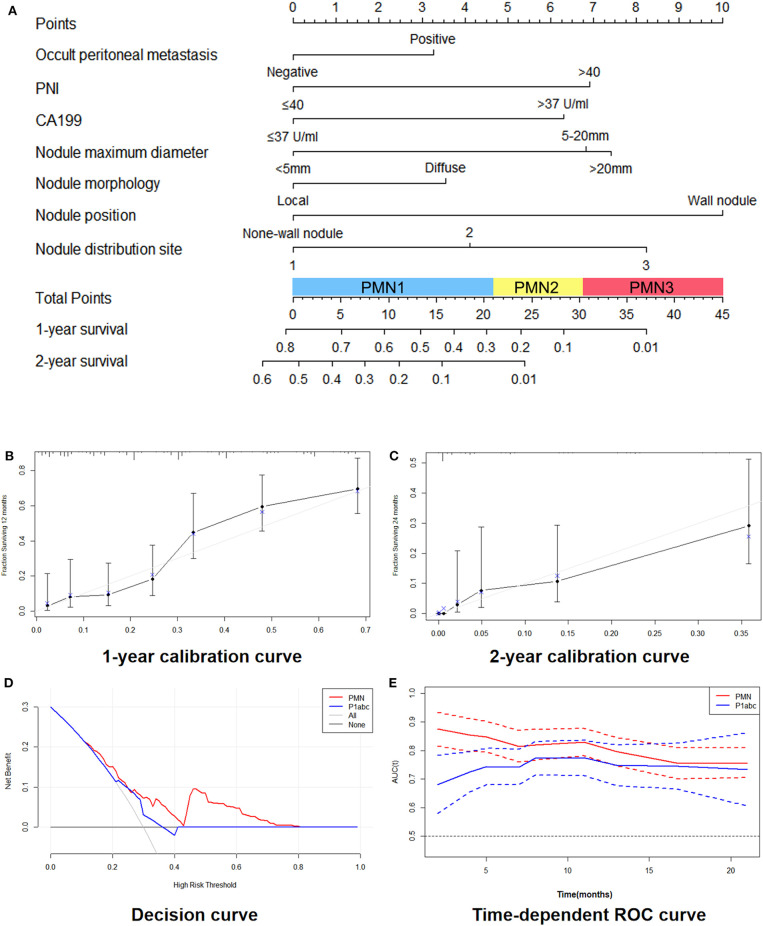
Development and performance of the nomogram. **(A)** Nomogram based on nodule signatures and clinical factors. **(B,C)**: 1-year calibration curves **(B)** and 2-year calibration curves **(C)** of the nomogram in the training cohort and validation cohorts. **(D)** Decision curve between the PMN and the P1abc. **(E)** Time-dependent ROC curve between the PMN and the P1abc.

We constructed the nomogram model according to the selected variables with a multivariate Cox analysis model in which each patient's total weighted score was calculated, and the corresponding PMN was obtained (Figure 1A). Figures 1B,C show the 1- and 2-year survival calibration curves of the training set, respectively. The decision curves in Figure 1D shows that the predictive threshold of the PMN was higher than that of the P1abc. In addition, a time-dependent receiver operating characteristic (ROC) curve was used to calculate the area under the curve (AUC) of the PMN and P1abc at different time points, and the AUC value of the PMN at each time point was higher than that of the P1abc (Figure 1E). b shows the 1- and 2-year survival calibration curves of the internal validation and external validation sets.

**Figure 2:**
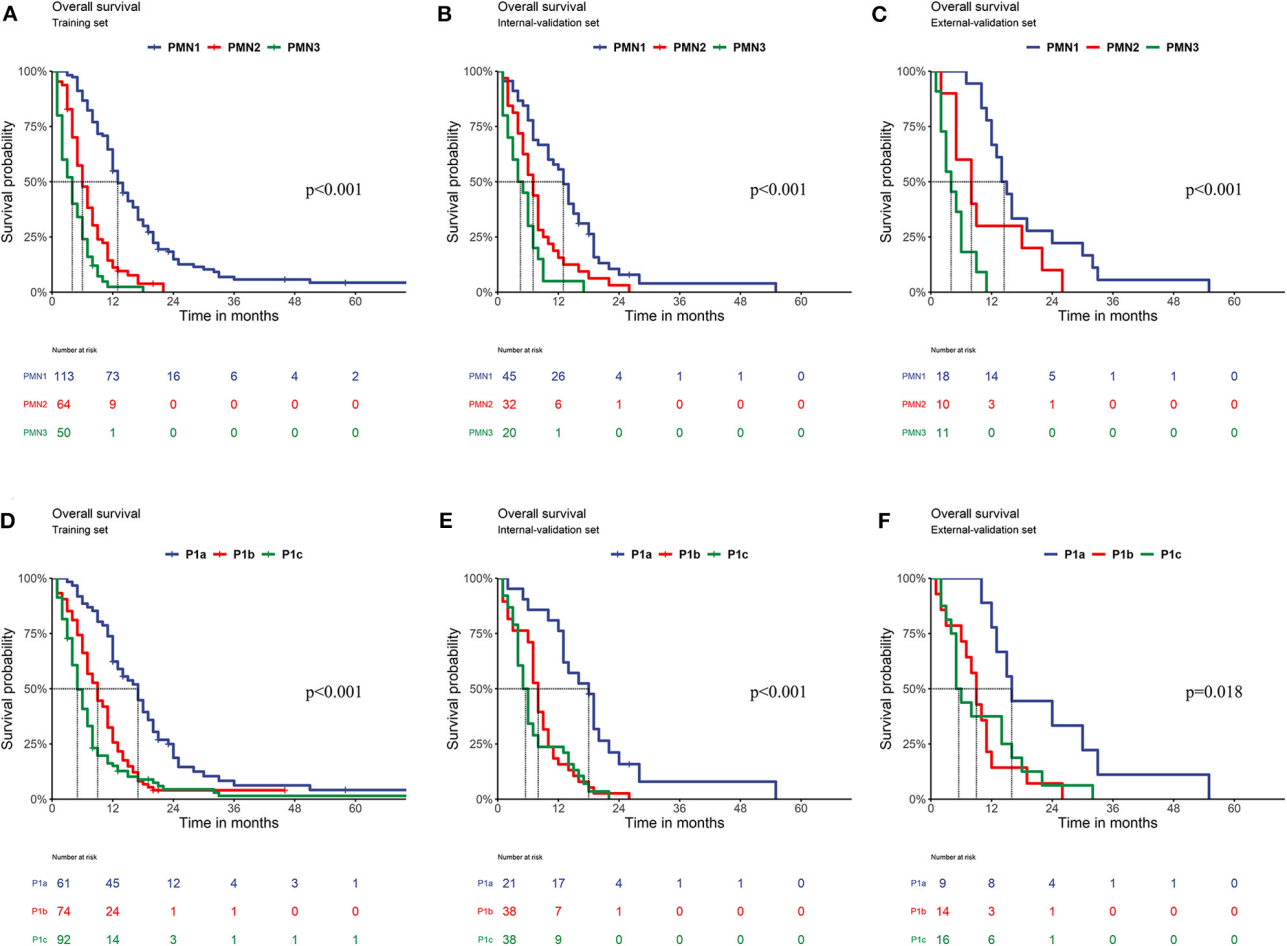
Comparison of OS. **(A)** Training set of the PMN (P_PMN1vs.PMN2_ < 0.001; P_PMN1vs.PMN3_ < 0.001; P_PMN2vs.PMN3_ < 0.001); **(B)** Internal validation set of the PMN (P_PMN1vs.PMN2_ = 0.001; P_PMN1vs.PMN3_ < 0.001; P_PMN2vs.PMN3_ = 0.041); **(C)** External validation set of the PMN (P_PMN1vs.PMN2_ = 0.039; P_PMN1vs.PMN3_ < 0.001; P_PMN2vs.PMN3_ = 0.036); **(D)** Training set of the P1abc (P_P1avs.P1b_ < 0.001; P_P1avs.P1c_ = *P* < 0.001; P_P1bvs.P1c_ = 0.022); **(E)** Internal validation set of the P1abc (P_P1avs.P1b_ < 0.001; P_P1avs.P1c_ < 0.001; P_P1bvs.P1c_ = 0.331). **(F)** External validation set of the P1abc (P_P1avs.P1b_ = 0.004; P_P1avs.P1c_ =0.022; P_P1bvs.P1c_ = 0.824).

In the training set, the survival curve showed that each PMN subgrade could be completely distinguished from the others. PMN1 patients had significantly better OS than PMN2 and PMN3 patients, and the OS of PMN2 patients was also significantly better than that of PMN3 patients (all *P* < 0.05). The survival curve of the P1abc showed that P1a patients had significantly better OS than P1b and P1c patients, and the OS of P1b patients was also significantly better than that of P1c patients (all *P* < 0.05). In both the internal validation and external validation sets, the subgroup survival analysis of PMN shows that the adjacent subgrades were well distinguished from one another. The OS of PMN1 patients was superior to that of both PMN2 and PMN3 patients (*P* < 0.05), and PMN2 patients had a significantly better OS than PMN3 patients. Among the P1abc, the OS of P1a patients was better than that of P1b and P1c patients, but the OS of P1b patients was close to that of P1c patients (all *P* > 0.05) (Figure 2). The results of the univariate and multivariate Cox analyses of each substage within the PMN are shown in Supplementary Table 3.

### Predictive Performance Comparisons

The PMN was superior to the P1abc in terms of the C-index (training set: PMN vs. P1abc = 0.747 vs. 0.671, *P* < 0.001; internal validation set: PMN vs. P1abc = 0.731 vs. 0.649, *P* = 0.007; external validation set: PMN vs. P1abc = 0.801 vs. 0.661, *P* = 0.032). The AIC analysis showed that the PMN had a better goodness of fit than the P1abc (training set: PMN vs. P1abc = 1853.4 vs. 1906.6, relative likelihood <0.001; internal validation set: PMN vs. P1abc = 658.31 vs. 668.95, relative likelihood = 0.004; external validation set: PMN vs. P1abc = 187.12 vs. 206.10, relative likelihood <0.001). The BIC analysis showed that the PMN had a better goodness of fit than the P1abc (training set: PMN vs. P1abc = 1880.8 vs. 1889.9; internal validation set: PMN vs. P1abc = 671.5 vs. 684.6; external validation set: PMN vs. P1abc = 200.4 vs. 201.1). Moreover, the PMN had better performance based on the likelihood ratio chi-square (likelihood ratio chi-square, training set: PMN = 110.93, P1abc = 40.906; internal validation set: PMN = 24.742, P1abc = 20.481; external validation set: PMN = 15.908, P1abc = 9.386) (Table 3).

**Table 3:** Comparison of the prognostic performance of different models.

	**Training set (*****n=*** **227)**		**Internal-validation set (*****n=*** **97)**		**External-validation set (*****n=*** **39)**	
	**PMN**	**P1abc**	**PMN**	**P1abc**	**PMN**	**P1abc**
Harrell's C index*	0.747 (0.711–0.782)	0.671 (0.633–0.708)	0.731 (0.672–0.792)	0.649 (0.584–0.714)	0.801 (0.733–0.869)	0.661 (0.544–0.769)
*P*-value**	–	<0.001	–	0.007	–	0.032
AIC†	1853.4	1906.6	658.31	668.95	187.12	206.10
BIC†	1880.8	1889.9	671.5	684.6	200.4	201.1
Relative likelihood††	–	<0.001	–	0.004	–	<0.001
Likelihood ratio chi-square‡	110.93	40.906	24.742	20.481	15.908	9.386

*AIC, Akaike information criterion*.

* *A higher Harrell's C index indicates higher discriminative ability*.

** *P-value of Harrell's C index (compare with the PMN grade)*.

† *Smaller AIC and BIC values indicate better optimistic prognostic stratification*.

†† *The relative likelihood could be interpreted as a p-value for the comparison of both AIC values (compared with the PMN grade)*.

‡ *A higher likelihood ratio chi-square score indicates better homogeneity*.

### Treatment Options Based on the PMN

**Figure 3:**
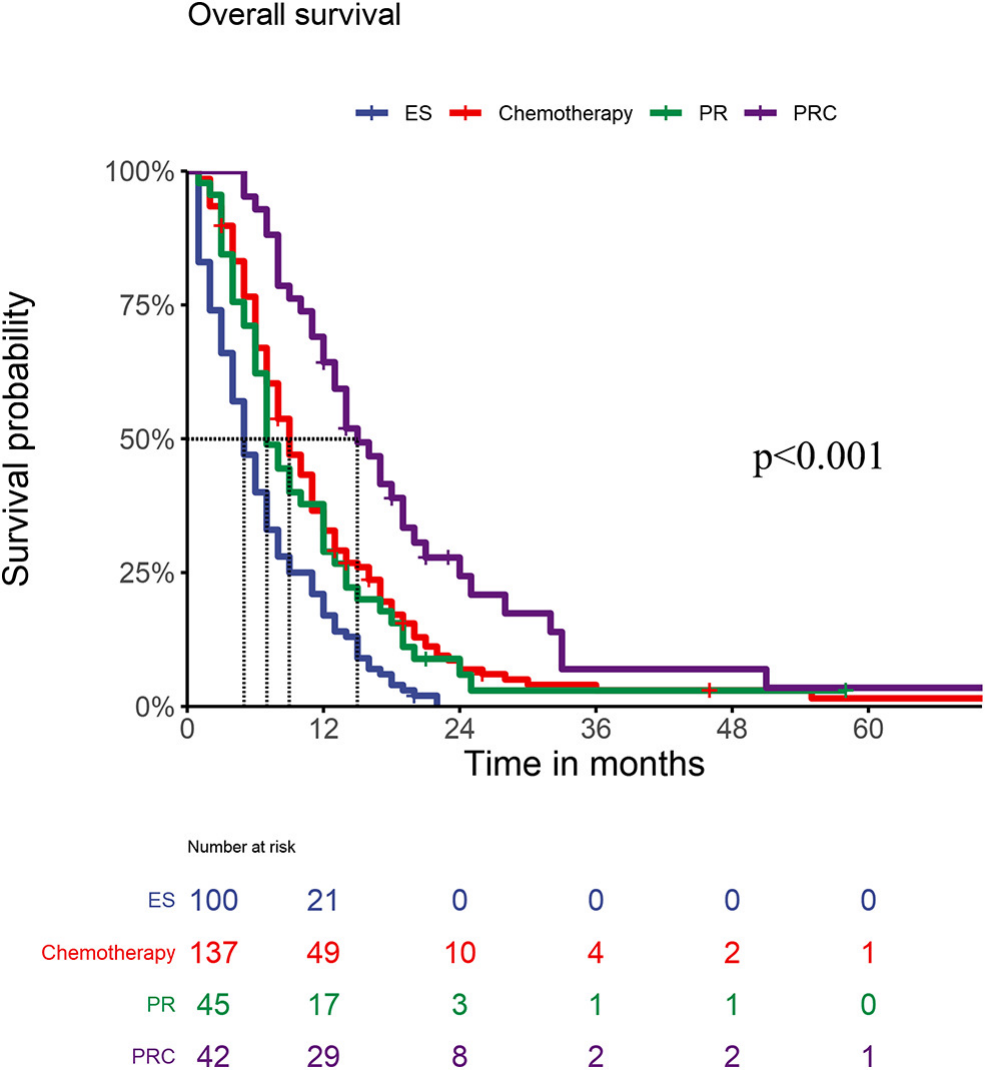
OS of PM patients within different treatments (P_ESvs.Chemotherapy_ < 0.001, P _ESvs.PR_ = 0.004, P_ESvs.PRC_ < 0.001; P_Chemotherapyvs.PR_ =0.519, P _Chemotherapyvs.PRC_ = 0.001; P _PRvs.PRC_ = 0.001).

Figure 3 shows the OS of patients with GCPM receiving different treatments. Patients with GCPM who received palliative resection and chemotherapy had a significantly better OS than those who received chemotherapy, palliative resection and exploratory surgery (all *P* < 0.05). No significant differences in OS were found between patients who received chemotherapy and those who received palliative resection (*P* = 0.519), but the OS of both of those patients was superior to the OS of patients who underwent exploratory surgery.

**Figure 4:**
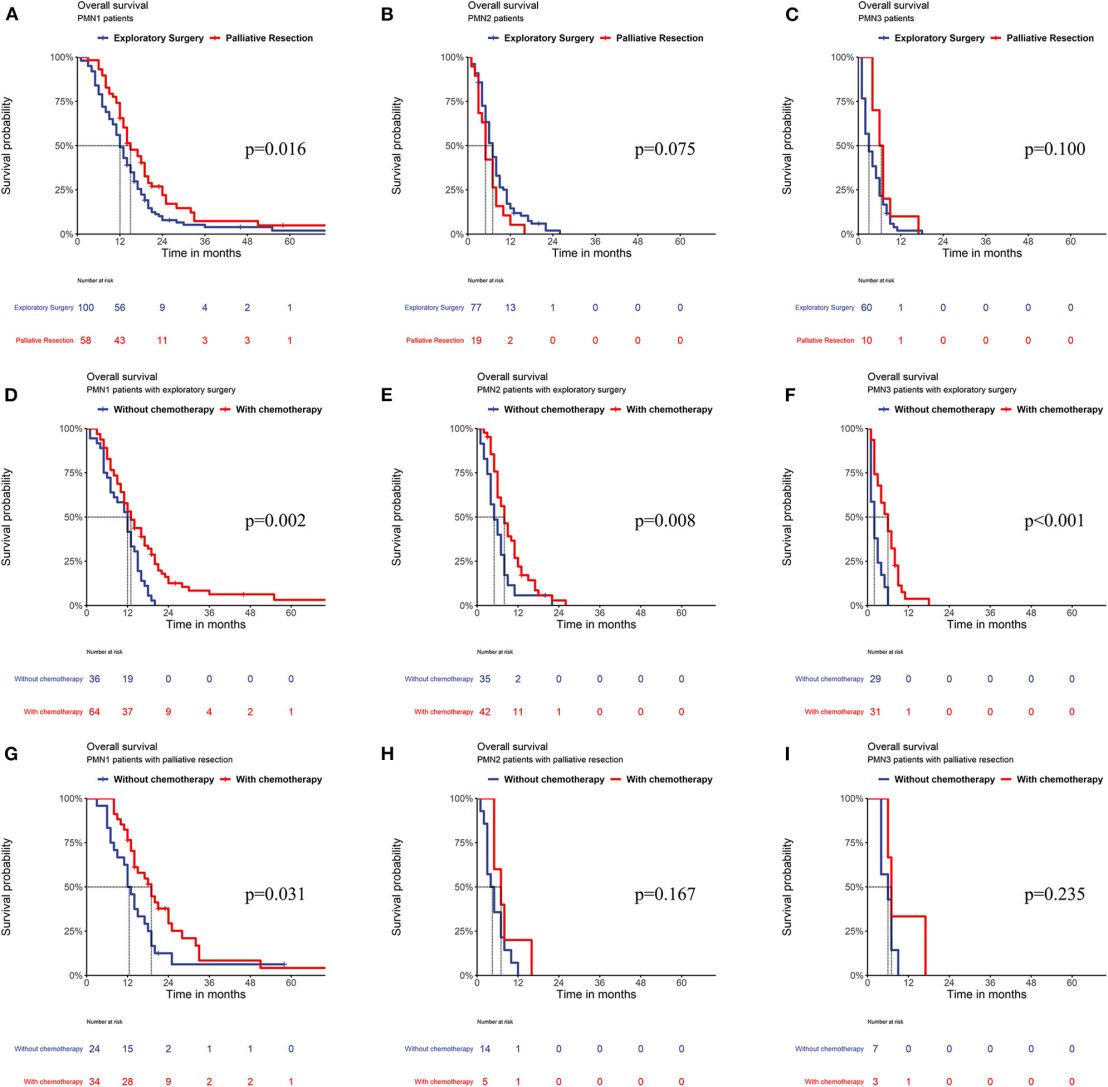
Relationship between type of surgery **(A–C)** and the PMN; **(D–F)** relationship between the PMN and exploratory surgery plus chemotherapy; **(G–I)** relationship between the PMN and palliative resection plus chemotherapy.

A stratified analysis of the surgical type showed that among the PMN1 patients, those who underwent palliative resection had a significantly better OS than those who underwent exploratory surgery (*P* = 0.016), while among PMN2 and PMN3 patients, the OS of patients who underwent palliative resection was similar to that of patients who underwent exploratory surgery (all *P* > 0.05). A further analysis of chemotherapy sensitivity showed that among PMN1, PMN2, and PMN3 patients undergoing exploratory surgery, chemotherapy could significantly improve the OS of these patients. However, only the OS of PMN1 patients was improved after chemotherapy among patients undergoing palliative resection (*P* = 0.031), and the OS of PMN2 and PMN3 patients who received chemotherapy was not significantly different from that of patients who did not (*P* > 0.05) (Figure 4).

## Discussion

Due to the high degree of malignancy, PM was considered to have prognostic characteristics different from non-metastatic GC. Therefore, accurate risk stratification for GCPM is very important not only for patients but also for clinicians. Based on the representative results of a translational study called PHOENIX (36), the 15th Japan Gastric Cancer Association (JGCA) guidelines published in 2017 revised the grading of PM. However, few studies have reported its ability to predict prognosis (14). Our study is here to fill in the gap by conducting the first comprehensive study to apply preoperative CT findings, the PNI and special nodule characteristics to explore the prognosis of GCPM. Our study found that indicators like preoperative imaging and nutritional indicators, the PNI, and CA19-9 are highly valuable and easily obtainable, and thus can provide helpful preoperative information for clinical surgeons to make individual prognostic assessments. We found that the maximum nodule diameter, nodule morphology, nodule position and number of nodule distribution sites were independent risk factors for OS, and that a lower PNI was associated with poor prognosis in GCPM. The significance of the PNI as a prognostic predictor has been revealed in various types of human cancers (37). Therefore, we believe that the systemic nutritional and immune status might be closely related to the prognosis of GCPM, indicating that prognostic assessments should be combined with basic immunology for the diagnosis of and therapeutic planning for GCPM. Based on these findings, our model, the peritoneal specificity model (PMN), combined preoperative imaging and nutritional indicators, the PNI, and CA19-9 with the conventional characteristics of peritoneal nodules, including size, morphology, range of spread and number of distribution sites. Our results confirm the value of these indicators, as is shown in detail in the following paragraphs. As CT is widely available in most medical centers because it is minimally invasive and relatively inexpensive, the indicators of the PNI and CA19-9, as part of routine clinical tests, can be easily obtained preoperatively for predicting the prognosis of GCPM. This means that our PMN is also highly applicable.

In previous studies (14), we have confirmed that P1abc was a relatively simple and effective assessment methods. However, compared to Gilly staging and the PCI, P1abc did not consider the size of the transferred nodules. In the subsequent analysis, P1abc staging fails to accurately recognize which type of patients can get chemotherapy benefit. In this study, we further improve staging by considering the morphology and size of peritoneal nodules and the preoperative CT and laboratory markers, thereby establishing an improved intraoperative model for predicting survival and deciding therapeutic schedules. PMN model not only can accurately classify the GCPM patient into different risk stratification, but can also, particularly for the therapy in urgent need, provide comprehensive plans of survival benefit for clinicians based on a large sample of cases.

Compared with the P1abc, whether the training set or internal and external validation sets was evaluated or not, the PMN had completely different survival curves. In addition, the PMN had better performance in terms of the C-index, prediction homogeneity, prediction accuracy and stability of the model than the P1abc. The calibration curve also showed that the PMN has good predictive accuracy. In addition, the decision curve analysis showed that the PMN can achieve better net benefits and had more clinical applicability than the P1abc. Therefore, in our opinion, the PMN can improve prognostic stratification for patients with GCPM and thus is more suitable for patients with GCPM than the P1abc.

The treatment of GCPM and intraoperative decision making are matters of great concern for clinical surgeons. A standard treatment for GCPM is, however, still in want. Although the REGATTA (5) study in 2016 noted that AGC patients did not experience survival benefits from gastrectomy with D1 lymph node dissection, that study was not designed for patients with GCPM alone but designed for AGC patients with a single incurable factor. Therefore, to date, no prospective study has been published to provide a therapeutic reference for GCPM. Our results show that the OS of PMN1 patients can significantly benefit from palliative resection and chemotherapy, while chemotherapy can improve the OS of PMN2 and PMN3 patients. PMN1 patients are in a relatively early stage of the disease with a lesser degree of distant metastasis. Thus, palliative resection might improve the OS of such patients. Palliative resection can remove visible tumor and reduce the potentially immunosuppressive tumor burden, eliminate the source of transfer, and improve the symptoms caused by gastric injury. Hoon et al. found that it is desirable for patients with GCPM with a low level of PM to undergo surgical resection of the primary tumors and metastatic lesions (38). Lee et al. (39) also confirmed that the survival of AGC patients with distant metastasis can benefit from gastrectomy due to a reduction in tumor burden. Furthermore, additional postoperative chemotherapy can kill free tumor cells *in vivo* as well as micrometastases. But the resection may not be the best choice for PMN2 and PMN3 patients. Because of the wide range of lesion spread in PMN2 and PMN3 patients, the invasiveness of the surgery itself and the poor condition of these patients, palliative or cytoreductive surgery can be a huge shock to the patients' bodies if the resection is incomplete. In such cases, surgery may even stimulate and increase the tumor burden. For surgeons who prefer an aggressive surgical strategy, we recommend avoiding unnecessary additional surgical interventions for PMN2 or PMN3 patients. Considering that open surgery leads to a high degree of trauma, we chose chemotherapy as a good alternative, a minimally invasive treatment compared with surgery, in an attempt to not only eradicate free tumor cells and micrometastases but also to reduce malignant ascites to the greatest extent possible.

Besides, compared with other nomograms from previous studies, the nomogram proposed by our study is more effective and applicable. In 2019, Dong et al. (40) published a study that included a total of 554 patients with AGC from different four centers. Patients' radiomics signatures (phenotypes of the primary tumor and peritoneal region) and Lauren types were applied for the construction of a nomogram. The nomogram, which consisted of radiomics signatures and clinical tumor factors, was built for predicting occult PM, and its AUC values in one training, one internal validation, and two external validation cohorts were all more than 0.920. However, the practical value is limited. The model was too complex, and required analysis of the corresponding CT images, making the nomogram too difficult to promote. Subsequently, they did not rely on the strong identification ability of the model to classify the patients and further analyzed the survival benefits and selection of treatment. Compared with other nomograms from previous studies (40, 41), our study combined preoperative clinical factors and intraoperative peritoneal nodule characteristics to build a nomogram that can classify the patients and suggest appropriate treatment options through analyzing the large sample. This nomogram can provide a reference for clinical decision making about appropriate treatment strategies for patients with GCPM. At the same time, this nomogram included the latest P1abc; thus, it is an effective supplement to previous studies.

We also developed and validated a predictive model based on parameters easily obtained preoperatively or intraoperatively in this study. This model can specifically predict the prognosis of GCPM and guide treatment decisions during surgery, and its accuracy, prediction and discrimination are superior to those of the P1abc. However, this study also has some limitations. First, because chemotherapy may change the features of the PM nodules, this study excluded patients who received chemotherapy before surgery; however, some scholars are still interested and concerned about this population. Second, some studies (27, 42) have suggested that there is no evidence to show the superiority of intraperitoneal chemotherapy over other forms of chemotherapy in terms of improving the prognosis of GCPM. Therefore, the chemotherapy regimen at our center mainly consists of systemic chemotherapy and supplementary intra-abdominal chemotherapy. However, additional multicenter data are needed to further explore whether this model is conducive to selecting proper patients for intra-abdominal chemotherapy. Finally, the main chemotherapy regimen for GCPM in this study comprised “5-fluoro” drugs. However, due to certain inherent biases of retrospective studies, Our conclusions may not be as authoritative as those of prospective studies, and thus could only serve as a reference for clinical decisions. Prospective studies should be given further attention and reported. We look forward to further validating the PMN model established in this study through a large sample with multicenter data in the future. In future work, patients should be stratified further to confirm the benefits of different chemotherapy regimens.

## Data Availability Statement

The raw data supporting the conclusions of this article will be made available by the authors, without undue reservation.

## Ethics Statement

The studies involving human participants were reviewed and approved by the Ethics Committee of FMUUH. Written informed consent from the patients was not required to participate in this study in accordance with the national legislation and the institutional requirements.

## Author Contributions

Q-YC, Z-YL, C-MH, and J-WX contributed conception and design of the study. Q-YC, Z-YL, WJ, Y-JZ, and C-HZ organized the database. Z-YL, QZ, C-MH, and J-WX performed the statistical analysis. Q-YC, Z-YL, and QZ wrote the first draft of the manuscript. PL, J-BW, J-XL, JL, L-LC, ML, R-HT, Z-NH, J-LL, H-LZ, and S-JQ wrote sections of the manuscript. All authors contributed to manuscript revision, read, and approved the submitted version.

## Conflict of Interest

The authors declare that the research was conducted in the absence of any commercial or financial relationships that could be construed as a potential conflict of interest.

## Supplementary Material

Supplementary Figure 1 Study cohort.

Supplementary Figure 2 Images of diffuse and local type metastasis, **(A)** local type; **(B)** diffuse type.

Supplementary Figure 3 1- and 2-year survival calibration curves of the internal validation set and external validation set.

Supplementary Table 1 Clinicopathologic description of the external validation set. SD, standard deviation; ASA, American Society of Anesthesiologists; LN, Lymph node; PNI, Prognostic nutritional index.

Supplementary Table 2 Cox regression analysis for training set of GCPM. NLR, Neutrophil-to-lymphocyte ratio; PLR, Platelet-to-lymphocyte ratio; PNI, Prognostic nutritional index; AGR, Albumin to globulin ratio; Evaluated by computed tomography: small, within the pelvic cavity; moderate, beyond the pelvic cavity.

Supplementary Table 3 Cox regression analysis for each substage within the PMN. NLR, Neutrophil-to-lymphocyte ratio; PLR, Platelet-to-lymphocyte ratio.

## Abbreviations

GCPM: gastric cancer with peritoneal metastasis
JCGC: Japanese Classification of Gastric Carcinoma
OS: overall survival
GC: gastric cancer
PCI: peritoneal Cancer Index
PM: peritoneal metastasis
LN: lymph node
ASA: American Society of Anesthesiologists
PMN: peritoneal metastasis nomogram
P1abc: 15th Japanese Classification of Gastric Carcinoma Staging Guidelines for Peritoneal Metastasis
PNI: prognostic nutritional index
ES: exploratory surgery
PR: palliative resection
PRC: palliative resection and chemotherapy
ROC: receiver operating characteristic
NLR: neutrophil-to-lymphocyte ratio
PLR: platelet-to-lymphocyte ratio.
