# Supplementary figures and images for: An Intraoperative Model for Predicting Survival and Deciding Therapeutic Schedules: A Comprehensive Analysis of Peritoneal Metastasis in Patients With Advanced Gastric Cancer
Source: Front Oncol. 2020 Sep 25;10:550526. doi: 10.3389/fonc.2020.550526 (PMC7546781; doi:10.3389/fonc.2020.550526)

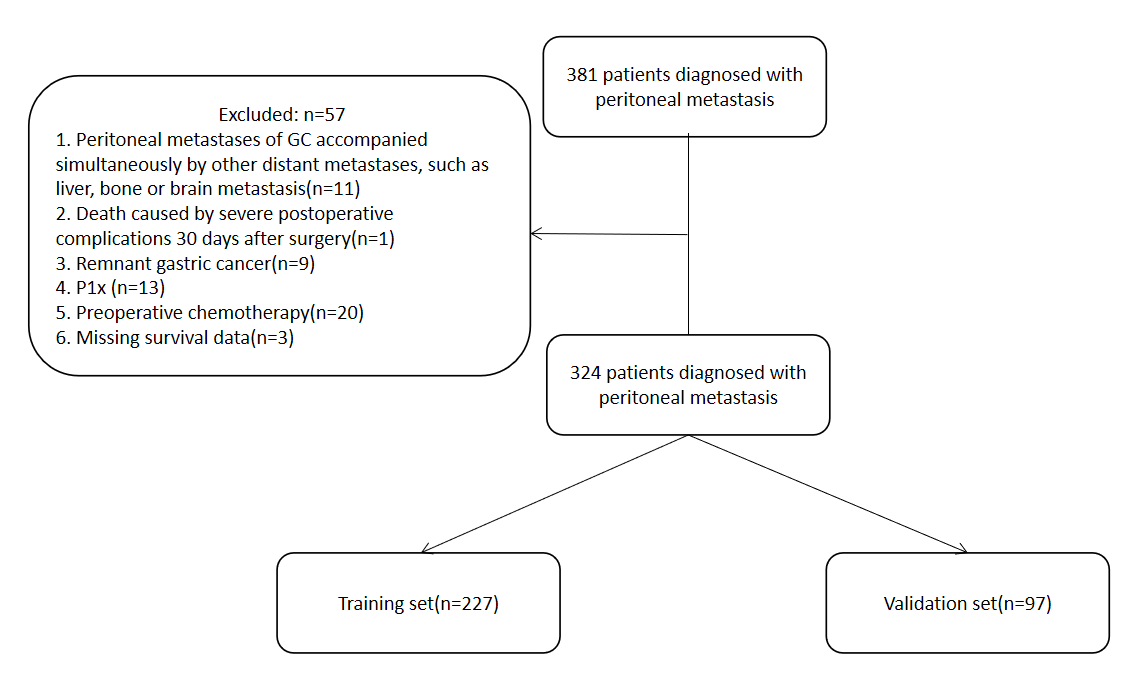

Supplement: Supplementary Figure 1 — Study cohort. [file Image_1.TIF]

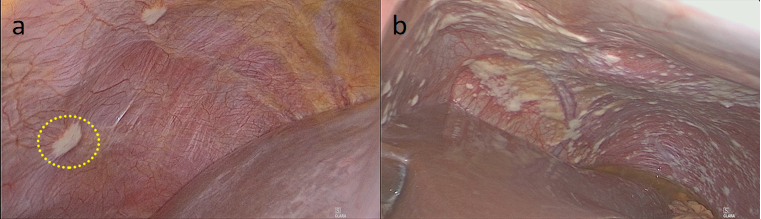

Supplement: Supplementary Figure 2 — Images of diffuse and local type metastasis, (A) local type; (B) diffuse type. [file Image_2.TIF]

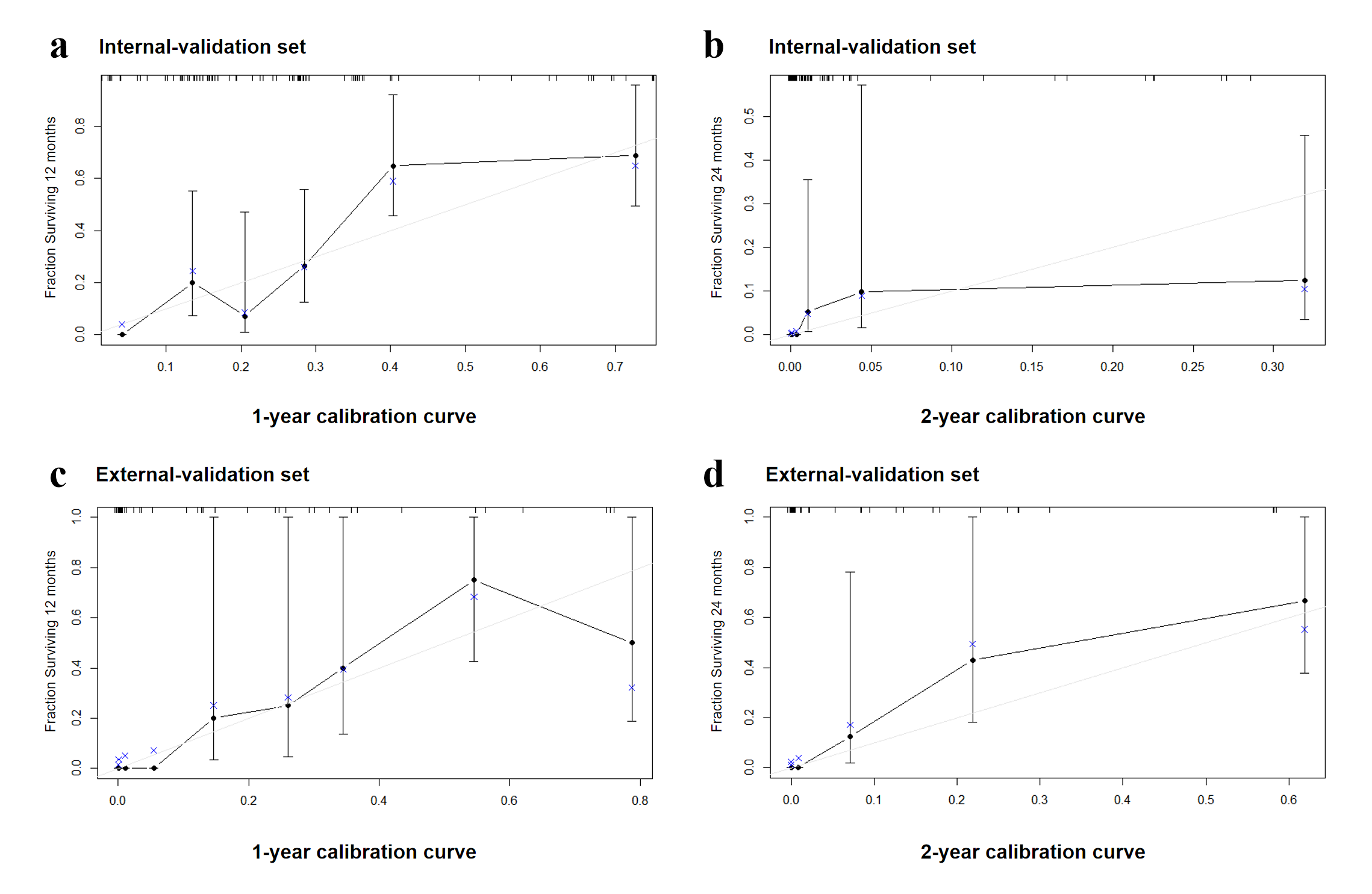

Supplement: Supplementary Figure 3 — 1- and 2-year survival calibration curves of the internal validation set and external validation set. [file Image_3.TIF]
