# Supplementary material for: An Intraoperative Model for Predicting Survival and Deciding Therapeutic Schedules: A Comprehensive Analysis of Peritoneal Metastasis in Patients With Advanced Gastric Cancer
Source: Front Oncol. 2020 Sep 25;10:550526. doi: 10.3389/fonc.2020.550526 (PMC7546781; doi:10.3389/fonc.2020.550526)
Supplement: Supplementary Table 1 — Clinicopathologic description of the external validation set. SD, standard deviation; ASA, American Society of Anesthesiologists; LN, Lymph node; PNI, Prognostic nutritional index. [file Table_1.docx]

Table S1 Clinicopathologic description of the external validation set

| Category | External-validation set | % |
| --- | --- | --- |
|  | (n=39) |  |
| **Age(years)** |  |  |
| ≤65 | 17 | 43.6 |
| >65 | 22 | 56.4 |
| **Sex** |  |  |
| Female | 14 | 35.9 |
| Male | 25 | 64.1 |
| **Occult peritoneal metastasis** | | |
| No | 23 | 59.0 |
| Yes | 16 | 41.0 |
| **cT** |  |  |
| cT2-3 | 3 | 7.7 |
| cT4 | 31 | 79.5 |
| cTx | 5 | 12.8 |
| **cN** |  |  |
| cNx | 4 | 10.3 |
| cN0 | 7 | 17.9 |
| cN+ | 28 | 71.8 |
| **Nodule maximum diameter** | | |
| <5mm | 12 | 30.8 |
| 5-20mm | 21 | 53.8 |
| >20mm | 6 | 15.4 |
| **Nodule morphology** | | |
| Local | 25 | 64.1 |
| Diffuse | 14 | 35.9 |
| **Nodule position** | | |
| none-wall | 9 | 23.1 |
| wall | 30 | 76.9 |
| **Number of nodule distribution site** | | |
| 1 | 21 | 53.8 |
| 2 | 12 | 30.8 |
| 3 | 6 | 15.4 |
| **P1abc** |  |  |
| P1a | 9 | 23.1 |
| P1b | 14 | 35.9 |
| P1c | 16 | 41.0 |
| **PNI** |  |  |
| ≤40 | 8 | 20.5 |
| >40 | 31 | 79.5 |
| **CA19-9, U/ml** |  |  |
| ≤37 | 24 | 61.5 |
| >37 | 15 | 38.5 |
| **Median survival, months** | 7(1-40) | |

Abbreviations: SD, standard deviation; ASA, American Society of Anesthesiologists ; LN, Lymph node; PNI, Prognostic nutritional index;
