# Supplementary material for: An Intraoperative Model for Predicting Survival and Deciding Therapeutic Schedules: A Comprehensive Analysis of Peritoneal Metastasis in Patients With Advanced Gastric Cancer
Source: Front Oncol. 2020 Sep 25;10:550526. doi: 10.3389/fonc.2020.550526 (PMC7546781; doi:10.3389/fonc.2020.550526)
Supplement: Supplementary Table 2 — Cox regression analysis for training set of GCPM. NLR, Neutrophil-to-lymphocyte ratio; PLR, Platelet-to-lymphocyte ratio; PNI, Prognostic nutritional index; AGR, Albumin to globulin ratio; Evaluated by computed tomography: small, within the pelvic cavity; moderate, beyond the pelvic cavity. [file Table_2.docx]

Table S2. Cox regression analysis for training set of gastric cancer of peritoneal metastasis

| Variable | Training set(n=227) | | | |
| --- | --- | --- | --- | --- |
|  | Univariate Model | | Reduced Multivariate Model | |
|  | Hazard ratio(95%CI) | P | Hazard ratio(95%CI) | P |
| **Age(years)** |  |  |  |  |
| ≤65 | Ref |  |  |  |
| >65 | 1.219(0.915-1.625) | 0.175 |  |  |
| **Sex** |  |  |  |  |
| Female | Ref |  |  |  |
| Male | 1.030(0.770-1.370) | 0.862 |  |  |
| **Occult peritoneal metastasis** | |  |  |  |
| No | Ref |  |  |  |
| Yes | 0.708(0.540-0.927) | **0.012** |  |  |
| **cT** |  | **0.023** |  | **0.006** |
| cT2-3 | Ref |  |  |  |
| cT4a | 0.728(0.481-1.101) | 0.132 | 0.775(0.505-1.191) | 0.245 |
| cT4b | 1.018(0.660-1.568) | 0.937 | 1.286(0.825-2.005) | 0.266 |
| cTx | 1.398(0.806-2.426) | 0.234 | 1.496(0.854-2.620) | 0.159 |
| **cN** |  | 0.223 |  |  |
| cN0 | Ref |  |  |  |
| cN+ | 1.298(0.911-1.850) | 0.149 |  |  |
| cNx | 1.498(0.918-2.444) | 0.106 |  |  |
| **Tumor location** | | 0.226 |  |  |
| Upper | Ref |  |  |  |
| Middle | 1.340(0.831-2.159) | 0.230 |  |  |
| Lower | 1.524(0.962-2.412) | 0.072 |  |  |
| Overlap | 1.127(0.649-1.956) | 0.671 |  |  |
| **Amount of ascites** | | **0.004** |  |  |
| None | Ref |  |  |  |
| Small | 1.091(0.760-1.565) | 0.637 |  |  |
| Moderate | 1.649(1.213-2.240) | **0.001** |  |  |
| **Nodule maximum diameter** | | **<0.001** |  | **<0.001** |
| <5mm | Ref |  |  |  |
| 5-20mm | 1.930(1.409-2.643) | **<0.001** | 1.807(1.279-2.553) | **0.001** |
| >20mm | 2.615(1.738-3.934) | **<0.001** | 2.276(1.470-3.524) | **<0.001** |
| **Nodule morphology** | |  |  |  |
| Local | Ref |  |  |  |
| Diffuse | 1.820(1.380-2.390) | **<0.001** |  |  |
| **Number of nodule distribution site** | | **0.030** |  | **0.011** |
| 1 | Ref |  |  |  |
| 2 | 1.388(1.02-1.889) | **0.037** | 1.471(1.057-2.047) | **0.022** |
| 3 | 1.403(0.906-2.174) | 0.129 | 1.772(1.129-2.782) | **0.013** |
| **P1abc** |  | **<0.001** |  | **<0.001** |
| P1a | Ref |  |  |  |
| P1b | 2.175(1.511-3.129) | **<0.001** | 1.908(1.310-2.780) | **0.001** |
| P1c | 2.991(2.111-4.238) | **<0.001** | 2.895(1.979-4.235) | **<0.001** |
| **SII** |  |  |  |  |
| ≤352 | Ref |  |  |  |
| >352 | 1.564(0.982-2.492) | 0.059 |  |  |
| **NLR** |  |  |  |  |
| ≤2 | Ref |  |  |  |
| >2 | 1.492(1.129-1.972) | **0.005** |  |  |
| **PLR** |  |  |  |  |
| ≤119 | Ref |  |  |  |
| >119 | 1.603(1.093-2.352) | **0.016** |  |  |
| **PNI** |  |  |  |  |
| ≤40 | Ref |  |  |  |
| >40 | 0.665(0.494-0.895) | **0.007** | 0.604(0.439-0.830) | **0.002** |
| **AGR** |  |  |  |  |
| ≤7 | Ref |  |  |  |
| >7 | 0.920(0.691-1.225) | 0.568 |  |  |
| **CEA,ng/ml** |  |  |  |  |
| <5 | Ref |  |  |  |
| ≥5 | 1.317(0.977-1.775) | 0.071 |  |  |
| **CA125,U/ml** |  |  |  |  |
| <35 | Ref |  |  |  |
| ≥35 | 1.445(1.091-1.914) | **0.010** |  |  |
| **CA199,U/ml** |  |  |  |  |
| ≤37 | Ref |  |  |  |
| >37 | 1.341(1.004-1.791) | **0.047** | 1.481(1.101-1.991) | **0.009** |

NLR, Neutrophil to lymphocyte ratio; PLR, Platelet to lymphocyte ratio; PNI, Prognostic nutritional index; AGR, Alb to globulin ratio; Evaluated by computed tomography: small, within the pelvic cavity; moderate, beyond the pelvic cavity.
