# Supplementary material for: An Intraoperative Model for Predicting Survival and Deciding Therapeutic Schedules: A Comprehensive Analysis of Peritoneal Metastasis in Patients With Advanced Gastric Cancer
Source: Front Oncol. 2020 Sep 25;10:550526. doi: 10.3389/fonc.2020.550526 (PMC7546781; doi:10.3389/fonc.2020.550526)
Supplement: Supplementary Table 3 — Cox regression analysis for each substage within the PMN. NLR, Neutrophil-to-lymphocyte ratio; PLR, Platelet-to-lymphocyte ratio. [file Table_3.doc]

Table S3 Cox regression analysis for each substage within PMN

| Variable | PMN1 | | PMN2 | | PMN3 | |
| --- | --- | --- | --- | --- | --- | --- |
|  | Univariate Model | | Univariate Model | | Univariate Model | |
|  | Hazard ratio(95%CI) | P | Hazard ratio(95%CI) | P | Hazard ratio(95%CI) | P |
| **Age(years)** |  |  |  |  |  |  |
| ≤65 | Ref |  | Ref |  | Ref |  |
| >65 | 1.230(0.861-1.757) | 0.255 | 1.141(0.739-1.762) | 0.551 | 1.578(0.952-2.615) | 0.077 |
| **Sex** |  |  |  |  |  |  |
| Female | Ref |  | Ref |  | Ref |  |
| Male | 1.116(0.789-1.578) | 0.535 | 1.117(0.726-1.718) | 0.614 | 0.952(0.573-1.581) | 0.850 |
| **cT** |  | 0.205 |  | 0.596 |  | 0.305 |
| cT2-3 | Ref |  | Ref |  | Ref |  |
| cT4a | 0.650(0.402-1.050) | 0.078 | 0.782(0.363-1.685) | 0.531 | 1.310(0.625-2.749) | 0.475 |
| cT4b | 0.907(0.560-1.469) | 0.693 | 0.874(0.403-1.894) | 0.733 | 1.75(0.858-3.572) | 0.124 |
| cTx | 0.910(0.391-2.113) | 0.826 | 1.239(0.494-3.107) | 0.648 | 2.198(0.824-5.864) | 0.116 |
| **cN** |  | 0.700 |  | 0.273 |  | 0.546 |
| cN0 | Ref |  | Ref |  | Ref |  |
| cN+ | 1.198(0.786-1.827) | 0.401 | 1.562(0.888-2.748) | 0.122 | 1.456(0.713-2.974) | 0.303 |
| cNx | 1.124(0.555-2.275) | 0.746 | 1.620(0.804-3.265) | 0.177 | 1.597(0.609-4.191) | 0.341 |
| **Tumor location** |  | 0.981 |  | 0.633 |  | 0.752 |
| Upper | Ref |  | Ref |  | Ref |  |
| Middle | 1.067(0.643-1.772) | 0.801 | 0.697(0.288-1.686) | 0.423 | 1.353(0.507-3.609) | 0.546 |
| Lower | 1.109(0.685-1.796) | 0.673 | 0.589(0.249-1.390) | 0.227 | 1.617(0.632-4.141) | 0.316 |
| Overlap | 1.062(0.604-1.867) | 0.834 | 0.700(0.262-1.876) | 0.479 | 1.427(0.425-4.792) | 0.565 |
| **Amount of ascites** |  | 0.564 |  | 0.758 |  | 0.670 |
| None | Ref |  | Ref |  |  |  |
| Small | 0.886(0.591-1.329) | 0.559 | 1.121(0.608-2.068) | 0.715 | 1.102(0.538-2.256) | 0.791 |
| Moderate | 1.138(0.769-1.683) | 0.518 | 1.195(0.747-1.912) | 0.457 | 1.289(0.723-2.297) | 0.389 |
| **Type of surgery** |  |  |  |  |  |  |
| Exploratory surgery | Ref |  | Ref |  | Ref |  |
| Palliative Resection | 0.669(0.475-0.943) | **0.022** | 1.535(0.921-2.561) | 0.100 | 0.606(0.308-1.192) | 0.147 |
| **SII** |  |  |  |  |  |  |
| ≤352 | Ref |  | Ref |  | Ref |  |
| >352 | 1.107(0.701-1.748) | 0.664 | 1.339(0.580-3.090) | 0.494 | 1.839(0.574-5.891) | 0.305 |
| **NLR** |  |  |  |  |  |  |
| ≤2 | Ref |  | Ref |  | Ref |  |
| >2 | 1.136(0.819-1.576) | 0.444 | 1.112(0.724-1.707) | 0.628 | 1.321(0.753-2.318) | 0.332 |
| **PLR** |  |  |  |  |  |  |
| ≤119 | Ref |  | Ref |  | Ref |  |
| >119 | 0.991(0.678-1.447) | 0.962 | 1.056(0.459-2.433) | 0.898 | 2.087(0.747-5.83) | 0.160 |
| **AGR** |  |  |  |  |  |  |
| ≤7 | Ref |  | Ref |  | Ref |  |
| >7 | 1.200(0.859-1.676) | 0.286 | 0.862(0.567-1.311) | 0.488 | 1.081(0.564-2.074) | 0.814 |
| **CEA,ng/ml** |  |  |  |  |  |  |
| <5 | Ref |  | Ref |  | Ref |  |
| ≥5 | 0.988(0.674-1.449) | 0.952 | 1.281(0.843-1.947) | 0.246 | 1.393(0.845-2.296) | 0.194 |
| **CA125,U/ml** |  |  |  |  |  |  |
| <35 | Ref |  | Ref |  | Ref |  |
| ≥35 | 0.901(0.650-1.249) | 0.532 | 1.201(0.771-1.872) | 0.417 | 1.530(0.777-3.010) | 0.218 |
| **Chemotherapy** |  |  |  |  |  |  |
| No | Ref |  | Ref |  | Ref |  |
| Yes | 0.885(0.636-1.231) | 0.467 | 0.646(0.428-0.976) | **0.038** | 0.409(0.245-0.683) | **0.001** |

NLR, Neutrophil to lymphocyte ratio; PLR, Platelet to lymphocyte ratio
