# Supplementary material for: An Intraoperative Model for Predicting Survival and Deciding Therapeutic Schedules: A Comprehensive Analysis of Peritoneal Metastasis in Patients With Advanced Gastric Cancer
Source: Front Oncol. 2020 Sep 25;10:550526. doi: 10.3389/fonc.2020.550526 (PMC7546781; doi:10.3389/fonc.2020.550526)
Supplement: Supplementary file 7 [file Data_Sheet_1.docx]

#AIC

setwd("E:/")

library(MASS)

library(foreign)

library(splines)

library(rms)

library(Hmisc)

lc<-spss.get('E:/OS-Nomogram.sav',use.value.labels=T,to.data.frame=T)

attach(lc)

coxm<-cph(Surv(OStime,OS)~p1abc,x=T,y=T,data=lc,surv=T)

scoxm<-step(coxm)

dd<-datadist(lc)

options(datadist="dd")

surv<-Survival(scoxm)

surv1<-function(x) surv(1*12,lp=x)

surv2<-function(x) surv(2*12,lp=x)

nom <- nomogram(scoxm,fun=list(surv1,surv2),lp=F,funlabel=c('1-year survival','2-year survival'),maxscale=10,fun.at=c(.95,.9,.8,.7,.6,.5,.4,.3,.2,.1,.01))

plot(nom, xfrac=.60)

print(nom)

set.seed(lc)

validate(coxm, B=100, dxy=TRUE)

rcorrcens(Surv(OStime,OS)~predict(scoxm))

survConcordance(formula=Surv(OStime,OS)~predict(scoxm),data=lc)

#1 year

coxm<-cph(Surv(OStime,OS)~OPM+pni+CA199+size+morphology+posi+range,x=T,y=T,data=lc,surv=T,time.inc=12)

cal<-calibrate(coxm, cmethod='KM', method='boot', u=12, m=4, B=100)

plot(cal)

#2 year

coxm<-cph(Surv(OStime,OS)~OPM+pni+CA199+size+morphology+posi+range,x=T,y=T,data=lc,surv=T,time.inc=24)

cal<-calibrate(coxm, cmethod='KM', method='boot', u=24, m=6, B=100)

plot(cal)

#C-index

library(MASS)

library(foreign)

library(splines)

library(rms)

library(Hmisc)

library(CsChange)

lc<-spss.get('E:/OS-Nomogram.sav',use.value.labels=T,to.data.frame=T)

attach(lc)

fit1=coxph(Surv(OStime,OS)~OPM+pni+CA199+size+morphology+posi+range)

fit2=coxph(Surv(OStime,OS)~p1abc)

CsChange(fit1,fit2,lc,nb=100,data=lc)

#Decision cureve

library(MASS)

library(foreign)

library(splines)

library(rms)

library(Hmisc)

library(rmda)

setwd("E:/")

lc<-spss.get('E:/OS-Nomogram.sav',use.value.labels=T,to.data.frame=T)

data.set<-lc

PMN<- decision_curve(OS~NP123,data = lc,family = binomial(link ='logit'), thresholds = seq(0,1, by = 0.01),confidence.intervals= 0.95,study.design = 'case-control',population.prevalence= 0.3)

P1abc<- decision_curve(OS~p1abc,data = lc,family = binomial(link ='logit'), thresholds = seq(0,1, by = 0.01),confidence.intervals= 0.95,study.design = 'case-control',population.prevalence= 0.3)

List<- list(PMN,P1abc)

plot_decision_curve(List,curve.names= c('PMN','P1abc'),cost.benefit.axis =FALSE,col = c( 'red', ' blue '),confidence.intervals =FALSE,standardize = FALSE)

summary(PMN,measure= 'NB')

#timeROC

install.packages("timeROC")

library(survival)

library(timeROC)

source("plotAUC.r")

data <- read.table('E:/OS-Nomogram.csv',stringsAsFactors = F,sep=",",head=T)

NP123 <-timeROC(T=data$Stime,delta=data$OS,marker=data$NP123,cause=1,weighting='marginal',ROC = TRUE,times=quantile(data$Stime,probs = seq(0,1,0.1)),iid=TRUE)

P1abc <- timeROC(T=data$Stime,delta=data$OS,marker=data$P1abc,cause=1,weighting='marginal',ROC = TRUE,times=quantile(data$Stime,probs = seq(0,1,0.1)),iid=TRUE)

plotAUCcurve(NP123,conf.int = TRUE,col="red")

plotAUCcurve(P1abc,conf.int = TRUE,col='blue',add=TRUE)

legend("topright",c("PMN","P1abc"),col=c('red','blue'),lwd=2)

print(NP123)

print(MPS)

#BIC

setwd("E:/")

library(splines)

library(rms)

library(Hmisc)

lc<-spss.get('E:/OS-Nomogram.sav',use.value.labels=T,to.data.frame=T)

coxm1<-cph(Surv(OStime,OS)~OPM+pni+CA199+size+morphology+posi+range,x=T,y=T,data=lc,surv=T)

coxm2<-cph(Surv(OStime,OS)~p1abc,x=T,y=T,data=lc,surv=T)

PMN<-BIC(coxm1)

P1abc<-BIC(coxm2)

result<-PMN/P1abc

PMN

P1abc

result
